# Multiblock Analysis Applied to Fluorescence and Absorbance Spectra to Estimate Total Polyphenol Content in Extra Virgin Olive Oil

**DOI:** 10.3390/foods10112556

**Published:** 2021-10-23

**Authors:** Natalia Hernández-Sánchez, Lourdes Lleó, Belén Diezma, Eva Cristina Correa, Blanca Sastre, Jean-Michel Roger

**Affiliations:** 1 Laboratorio de Propiedades Físicas y Técnicas Avanzadas en Agroalimentación (LPF_Tagralia), Escuela Técnica Superior de Ingeniería Agronómica, Alimentaria y de Biosistemas, Universidad Politécnica de Madrid, Av. Puerta de Hierro, 2–4, 28040 Madrid, Spain; lourdes.lleo@upm.es (L.L.); belen.diezma@upm.es (B.D.); evacristina.correa@upm.es (E.C.C.); 2 Applied Research Department, IMIDRA, Finca El Encín, Alcalá de Henares, 28805 Madrid, Spain; blanca.esther.sastre@madrid.org; 3 Chemhouse Research Group, ITAP, Université de Montpellier, Irstea, Montpellier SupAgro, 34060 Montpellier, France; jean-michel.roger@irstea.fr

**Keywords:** antioxidants, chemometrics, spectroscopy

## Abstract

A fast and easy methodology to estimate total polyphenol content in extra virgin olive oil was developed by applying the chemometric multiblock method sequential and orthogonalized partial least squares (SO-PLS) in order to combine front-face emission fluorescence spectra (270 nm excitation wavelength) and absorbance spectra. The hypothesis of this work stated that inner-filter effects in fluorescence spectra that would reduce the estimation performance of a single block model could be overcome by incorporating the absorbance spectral information of the compounds causing them. Different spectral preprocessing algorithms were applied. Double cross-validation with 50 iterations was implemented to improve the robustness of the obtained results. The PLSR model on the single block of fluorescence raw spectra achieved an RMSEP of 177.11 mg·kg^−1^ as the median value, and the complexity of the model was high, as the median value of latent variables (LVs) was eight. Multiblock SO-PLS models with pretreated fluorescence and absorbance spectra provided better performance, although artefacts could be introduced by transformation. The combination of fluorescence and absorbance raw data decreased the RMSEP median to 134.45 mg·kg^−1^. Moreover, the complexity of the model was greatly reduced, which contributed to an increase in robustness. The median value of LVs was three for fluorescence data and only one for absorbance data. Validation of the methodology could be addressed by further work considering a higher number of samples and a detailed composition of polyphenols.

## 1. Introduction

Olive oil polyphenols are antioxidant molecules included in the European Regulation No 432/2012 [1] that establishes the list of health claims which may be made on foods. The corresponding claim states that the olive oil polyphenols contribute, among other health benefits, to the protection of blood lipids from oxidative stress [2]. Additionally, polyphenols are involved in quality stability of olive oil [3]. The loss of freshness of olive oil involves the degradation of polyphenols, which, by oxidizing first, prevents the oxidation of other compounds in the olive oil.

The traditional method used to determine the global content of polyphenols is a colorimetric assay where the Folin–Ciocâlteu reagent is added to an extract of phenolic compounds obtained from the olive oil sample [4]. This method requires a calibration curve obtained with a solution of an antioxidant used as a standard, caffeic acid. In 2009, the International Olive Oil Council (COI) published a method for the determination of biophenols in olive oils by high-performance liquid chromatography (HPLC) [5]. The method is based on direct extraction of the biophenolic minor polar compounds from olive oil by means of a methanol solution and subsequent quantification by HPLC with the aid of an ultraviolet (UV) detector at 280 nm. Spectral recording for identification purposes is facilitated by using a photodiode detector with a spectral range from 200 nm to 400 nm. Syringic acid is used as the internal standard. The content of the natural and oxidised oleuropein and ligstroside derivatives, lignans, flavonoids, and phenolic acids is expressed in mg of tyrosol per kg of oil. Both methods, the COI method and the Folin–Ciocâlteu method, are time-consuming and require a cumbersome preparation of the oil sample using diverse chemical reagents and solutions. In addition, the COI method has to be performed by a specialist using the HPLC–UV/visible equipment. 

Fluorescence spectroscopy is a technique with great potential to provide a simpler method where no reagents and solutions would be required, and a nonexpert would be able to use the devoted equipment. Squeo et al., (2019) [6] explored the feasibility of direct front-face fluorescence excitation–emission matrices (EEMs) coupled with chemometrics for developing multivariate models for determination of total phenolic content (TPC). EEMs were obtained from olive oil samples without any treatment by recording the emission spectra from 260 to 700 nm with the excitation wavelengths ranging between 250 and 500 nm, at 10 nm steps. As a result, 26 spectra were required for each oil sample, which involved long-time experiments. The partial least squares (PLS) analysis of the unfolded entire EEMs for the estimation of TPC achieved a coefficient of determination *R*^2^ = 0.951 and a ratio of performance to deviation RPD = 4.0. However, the olive oil samples formed two groups characterized by distinctly different TPC values and limited variability within each group, which the authors assumed to be a cause of the model success. Regression analysis for the separate groups did not provide successful results.

The fluorescence phenomenon is provoked by a diversity of olive oil compounds that are related to its quality, which is an advantage for its evaluation. However, such an advantage might become a hindrance for the estimation of polyphenols due to the inner-filter effects. For the first-order inner filtering, olive oil compounds along the optical path length absorb part of the light that otherwise would excite polyphenols. Thus, the intensity of the excitation light for polyphenols is lower than that emitted by the source. As for the second-order inner effect, part of the light emitted by the polyphenols after being excited is reabsorbed before reaching the detector. Consequently, the fluorescence signal detected is decreased [7]. Front-face arrangement of the optical fibers diminishes to a great extent such a problem as the path length is extremely reduced. Notwithstanding this improvement, the hypothesis stated in the present work is that there are remaining inner-filter effects that could be overcome by incorporating information on the content of olive oil compounds that would cause such filtering effects.

EEMs provide the signal of a variety of fluorescence compounds in olive oil that are excited at different wavelengths. In previous works where front-face EEMs were obtained by exciting at a wavelength each time [6,8,9,10], when the olive oil samples were excited at the characteristic excitation band of the phenolic compounds, i.e., around 270 nm [9], two emission regions appeared. The emission region from approximately 300 to 380 nm corresponded to polyphenols, and the emission region from approximately 600 to 700 nm was ascribed to chlorophyll and its derivatives.

In olive oil pheophytin-*a* and pheophytin-*b* comprise the main chlorophyll fractions. During the oil extraction process, the released acids promote the pheophytinization of the chlorophylls, whereby the magnesium ion Mg^2+^ of the porphyrin ring is replaced by H^+^. The pheophytin content (*a* + *b*) accounts for more than 90% of the chlorophyll fraction [11]. Therefore, throughout the paper, they are referred to as pheophytins instead of chlorophylls. In addition, EEMs showed that compounds related to oxidation processes were excited by wavelengths that were within the range of the polyphenol emission (300 to 380 nm) [9,10]. A major issue of the analysis of olive oil using fluorescence spectroscopy is that it only provides information on fluorescent compounds. However, other compounds that are not fluorophores are also a potential cause of inner-filter effects, such as the carotenoid pigments. In olive oil, lutein is the main carotenoid pigment. Absorbance spectral data are proposed in the present work as a source of information on such compounds that might reduce the remaining filtering problems.

The chemometric multiblock method named sequential and orthogonalized partial least squares (SO-PLS) [12,13] was applied to simultaneously handle both spectral sources, fluorescence and absorbance spectra, as well as to model the relationships with the total polyphenol content. Double cross-validation was implemented during PLS and SO-PLS computation in order to improve the robustness of the obtained results [14]. 

Summarizing, the aim of the present work was to develop a fast and easy methodology to estimate total polyphenols content, whereby the information of front-face emission fluorescence spectral data was combined with the information of absorbance spectral data. 

## 2. Materials and Methods

### 2.1. Samples

Forty-eight extra virgin olive oil (EVOO) samples were used in this study. The extraction and quantification of the polyphenolic fraction were carried out according to the method described by Vázquez-Roncero et al. (1973) [4]. After methanol extraction, the quantification was carried out based on the formation of a colored complex with the Folin–Ciocâlteau reagent and spectrophotometrically determined at an absorption wavelength of 725 nm. The absorbance was compared to that generated by a known amount of the standard caffeic acid, and the results were expressed in ppm of caffeic acid (mg caffeic acid/kg of oil).

The global quantification of pigments (carotenoids and chlorophylls) was based on the dissolution of an olive oil sample in cyclohexane and the spectrophotometric reading at 472 and 670 nm, respectively [15]. The carotenoid fraction was quantified by measuring the absorbance at 470 nm, which corresponds to lutein (70% of the total carotenoid pigments), and the chlorophyll fraction was quantified by measuring the absorbance at 670 nm, which corresponds to pheophytin, the major component of this fraction. 

**Figure 1:**
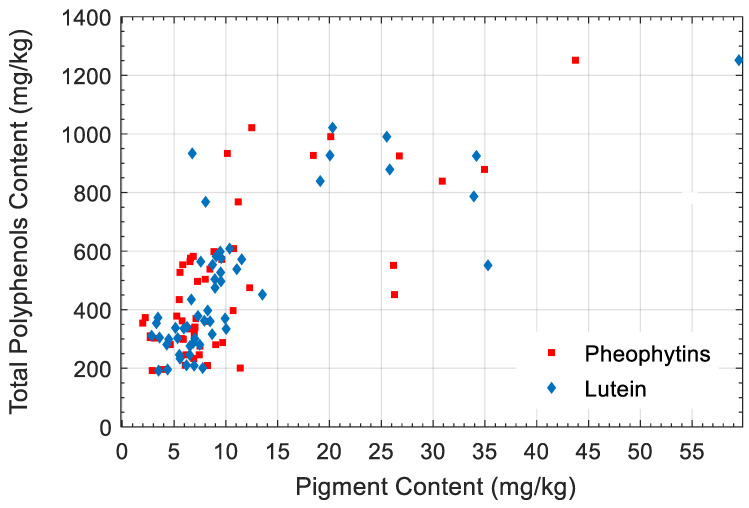
Scatter plot of the total polyphenol content and the pheophytin and lutein pigment content.

Table 1 summarizes the basic statistics for the total polyphenols, the pheophytins, and the lutein content. Regarding polyphenols, samples showed a wide range of variability from 192 to 1251 mg·kg^−1^, which is appropriate for estimation model development. Figure 1 shows the scatter plot of the total polyphenol content (*y*-axis) and the pheophytin (*R*^2^ = 0.45) and lutein content (*R*^2^ = 0.58) (*x*-axis), which illustrates their poor relationship as the pigment content in olive oil is a trait highly related to the cultivar, with no direct relationship with polyphenol content.

### 2.2. Fluorescence Spectral Data

A high-power Xenon light source HPX-2000 (Ocean Optics Inc, Orlando, FL, USA), with a compartment to accommodate optical filters, was used. A bandpass filter (OptoSigma^®^, Santa Ana, CA, USA) centered at 270 nm with a 10 nm bandwidth was set to excite the phenolic compounds in the samples, which produces an emission spectrum due to the fluorescence phenomenon. 

Fluorescence spectra were acquired using a QE *Pro* spectrometer (Ocean Optics Inc., Orlando, FL, USA). Integration time was set to 10,000 ms, and three repetitions per measurement were averaged. Wavelengths of the emission spectra included in the study ranged from 304 nm to 781.8 nm with a spectral resolution of 0.79 nm. 

Measurements were carried out in a front-face setup in order to minimize inner-filter effects. Quartz cuvettes were filled with the oil samples without prior preparation. 

### 2.3. Transmitance Spectral Data

A desktop spectrophotometer (Hamamatsu Photonics K.K., Shizuoka, Japan) was used to obtain transmittance spectra. Transmitance spectral data (T(λ)) from 348.4 nm to 722.1 nm, with a spectral resolution of 0.77 nm, were then transformed to absorbance values as A(λ) = log (1/T(λ)) to comply with the Bouguer–Beer–Lambert law.

Quartz cuvettes with 10 mm optical path length containing oil samples were illuminated by a halogen lamp UV/Vis (L10290, Hamamatsu Photonics K.K., Shizuoka, Japan). Exposure time was 20 ms and the number of counts averaged was 5.

### 2.4. Chemometrics

Several preprocessing methods were applied to both fluorescence and absorbance spectra, as reported in Table 2. Raw spectra and preprocessed spectral data entered the analysis for estimation of total polyphenol content.

Partial least squares regression (PLS) was applied to each set of preprocessed fluorescence emission spectra with regard to total polyphenol content. PLS builds *K* latent variables which are linear combinations of the columns of *X*, in order to maximize the square covariance between these new variables and the response *Y* to be predicted. Predictions of *Y* values are made using Equation (1).

(1) $$\hat{Y}=X^{\;}b,$$

where *b* is the vector of *b*-coefficients of the PLS. These *b* coefficients have the same dimension as a spectrum and can be analyzed as such.

Preprocessed fluorescence and absorbance spectra were combined in order to assess the capability of the fluorescence spectra to estimate total polyphenol content. Each spectral dataset was considered as an independent block and entered the chemometric multiblock method SO-PLS. SO-PLS is a multivariate linear projection method based on PLS regression that handles all the data blocks at the same time and models the relationships with the response to be estimated [12]. SO-PLS is a sequential use of PLS regression in combination with orthogonalization which focuses on the incremental contributions of each new block. SO-PLS builds *K*_1_, *K*_2_, …, *K_b_* latent variables which are linear combinations of the columns of the blocks *X*_1_, *X*_2_, …, *X_b_*. Predictions of *Y* values are made using Equation (2).

(2) $$\hat{Y}=X_{1}{}^{\;}b_{1}+X_{2}{}^{\;}b_{2}+\ldots +X_{b}{}^{\;}b_{b},$$

where *b*_1_, *b*_2_, and *b_b_* are the vectors of regression coefficients of the SO-PLS. As with classical PLS, the regression coefficients can be analyzed as spectra.

Whatever the calibration method (PLS or SO-PLS), the potential of the model was evaluated by means of a double cross-validation. According to Filzmoser et al., (2009) [14], for a small number of samples, the (frequently random) split into a calibration set, which is used for model building, and a test set for model evaluation can be performed several times to obtain a reasonable estimation of the optimum model complexity, as well as of the range of the prediction errors for new samples.

Fifty iterations were performed in the present work. For each iteration the scheme was as follows:

A calibration set with two-thirds of the samples (2/3 *N*) and a test set with one-third (1/3 *N*) were randomly selected;

A leave-one-out cross-validation on the calibration set with 1–20 latent variables (LV) was performed;

The number of latent variables corresponding to the smallest standard error of cross-validation (SECV) was chosen (LV_opt_);

A model with LV_opt_ latent variables was calibrated on the whole calibration set;

This model was applied to the test set.

The root-mean-squared error of prediction (RMSEP) was calculated using Equation (3).

(3) $$\mathrm{RMSEP}=\sqrt{\frac{\sum_{i=1}^{i=N}{\left(y_{i}-{\hat{y}}_{i}\right)}^{2}}{N}},$$

where *N* is the number of samples estimated, $y_{i}$ is the actual value of the response, and ${\hat{y}}_{i}$ is the predicted value of the response.

This method produced a series of RMSEPs and a series of LV_opt_ values (50 values corresponding to the 50 iterations). For simple PLS, each LV_opt_ is a single value. For SO-PLS, each LV_opt_ is a vector of the same size as the number of blocks. Boxplots of LV_opt_ and RMSEP values illustrate the estimation of the model complexities and performances.

## 3. Results and Discussion

### 3.1. Main Features of Fluorescence Spectra

**Figure 2:**
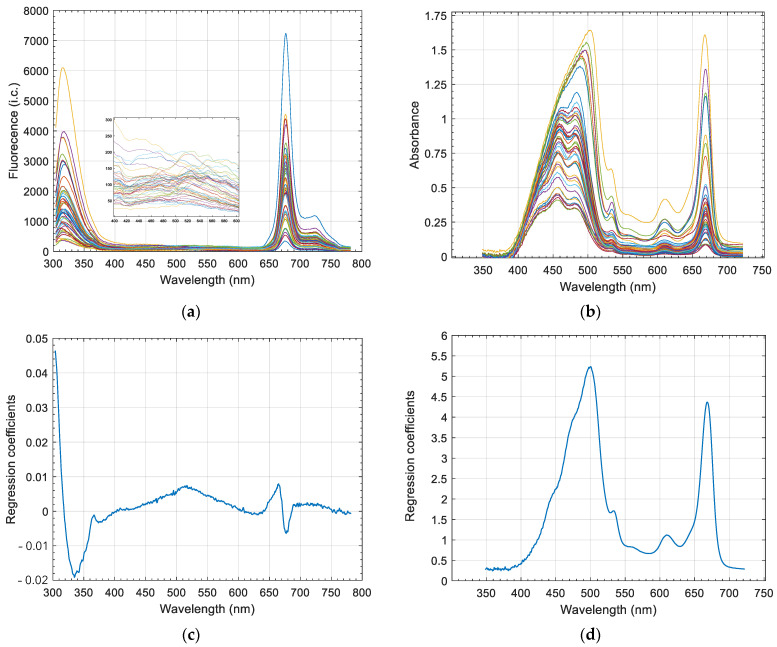
(**a**) Fluorescence emission spectra of EVOO at 270 nm excitation wavelength (*n* = 48 samples); (**b**) absorbance spectra for the same samples; (**c**) regression coefficients of the SO-PLS multiblock model for the fluorescence raw data block (three LVs); (**d**) regression coefficients of the SO-PLS multiblock model for the absorbance raw data block (one LV).

The excitation wavelength at 270 nm was selected as it produces the fluorescence emission of the polyphenols. Such emission occurs within the region from 300 nm to 400 nm. The fluorescence emission spectra obtained from the EVOO samples consistently showed such a region (Figure 2a). However, two additional peaks appeared centered at about 670 nm and 720 nm (Figure 2a), which were ascribed to the signal arising from pheophytins *a* and *b* according to the literature [8]. A signal around 720 nm appeared as a consequence of the reabsorption of the light at 670 nm by the pheophytins themselves and the reemission [7]. 

As light at 270 nm did not produce direct excitation of pheophytins, the excitation was produced by the light emitted by the polyphenols. This supposition is consistent with our previous results, with a spectral range similar to the present research, where 3D front-face spectra revealed that the range of wavelengths that excited the pheophytins included the wavelengths around 400 nm of polyphenol emission [9,10]. Such a study also showed that other olive oil compounds, i.e., oxidation products and tocopherols, would also be excited by the light emitted by the polyphenols.

In accordance with the hypothesis of the present work, fluorescence spectra revealed that the pigments contained in the samples would absorb part of the light emitted by the polyphenols, with the signal being lower than expected, which would induce a deviation in the relationship between the polyphenol content and the intensity of their signal detected by the spectrometer.

**Figure 3:**
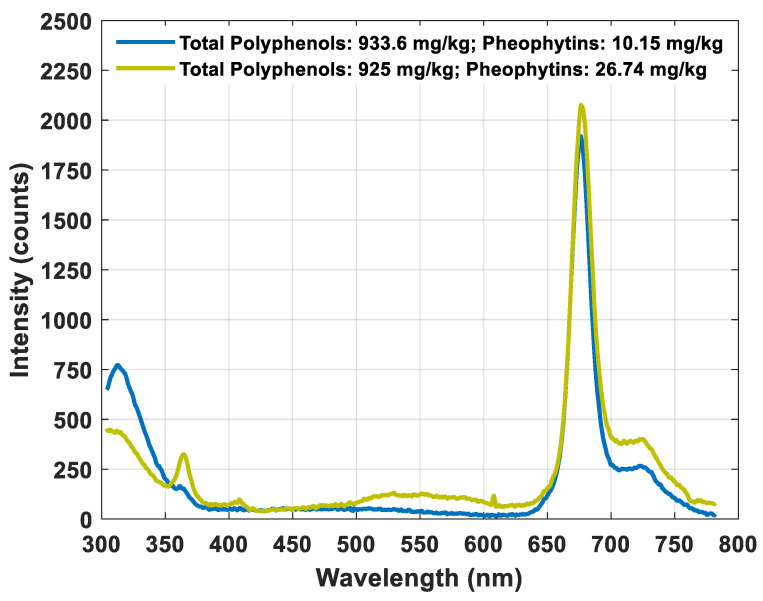
Example of fluorescence spectra of two samples with similar total polyphenol content and different pheophytin content.

A comparison between fluorescence spectra from samples with similar polyphenol but different pheophytin contents was carried out to illustrate such phenomena. Figure 3 shows the fluorescence spectra of two samples with 933.6 mg·kg^−1^ and 925 mg·kg^−1^ total polyphenols and 10.15 mg·kg^−1^ and 26.74 mg·kg^−1^ pheophytin content, respectively. Despite having almost the same content, the height of the peaks corresponding to polyphenols was clearly different. The sample with a higher content of pheophytins showed lower polyphenol emission intensity. This figure illustrates the suggested deviation. Moreover, such a sample showed an emission band from 500 to 600 nm, probably related to the presence of oxidation products and tocopherols, which would also be a consequence of the excitation produced by the emission of the polyphenols. The noticeable peak at 354 nm was probably associated with vanillic acid, a type of phenolic compound, which was probably directly excited by the light beam at 270 nm [17]. Other compounds, such as luteins, would reabsorb the light emitted by the polyphenols contributing to a reduction in the peak.

In view of these results, absorbance spectra from 348.4 nm to 722.1 nm were included in further analysis to integrate the influence of such absorbing compounds on the fluorescence spectral response of the polyphenols.

### 3.2. Main Features of Absorbance Spectra

Absorbance spectra from 348.4 nm to 722.1 nm showed a succession of overlapping absorbance peaks within the region between 400 and 700 nm (Figure 2b). The pattern was consistent with the characteristic peaks ascribed to the pigments contained in olive oil. According to Domenici et al., (2014) [18], for pheophytin-*a*, the main absorption regions are located at about 414 and 670 nm; for pheophytin-*b*, they are located at 437 and 657 nm; for lutein, they are located at 432, 455 and 486 nm; for β-carotene, they are located at 462 and 490 nm. 

Before integrating these spectra into the SO-PLS model for polyphenols content estimation, PLSRs with double cross-validation were applied to the raw absorbance in order to assess their reliability estimating actual content of the major pigments, pheophytins, and lutein.

For pheophytins, the model with three LVs (median value from double cross-validation) achieved an *R*^2^ of 0.94 and an RMSEP of 2.43 mg kg^−1^; for lutein, the model with nine LVs (median value from double cross-validation) achieved an *R*^2^ of 0.98 and an RMSEP of 1.15 mg kg^−1^, which was revealed to be excellent in terms of estimation performance. Subsequently, these absorbance spectra were included in further multiblock analysis.

### 3.3. Models for Polyphenol Estimation

**Figure 4:**
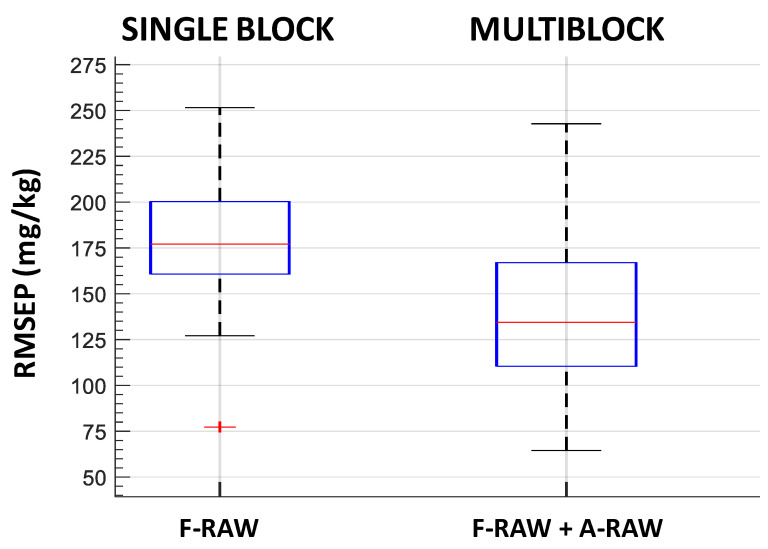
RMSEP boxplots of 50 iterations of the double cross-validation: on the left for PLSR model with fluorescence raw data; on the right for multiblock SO-PLS model with fluorescence and absorbance raw data. Bottom and top edges of the blue box are the 25th and 75th percentiles, respectively; the central mark is the median; whiskers extend to the most extreme data points not considered outliers; the ‘+’ symbol plots outliers.

**Figure 5:**
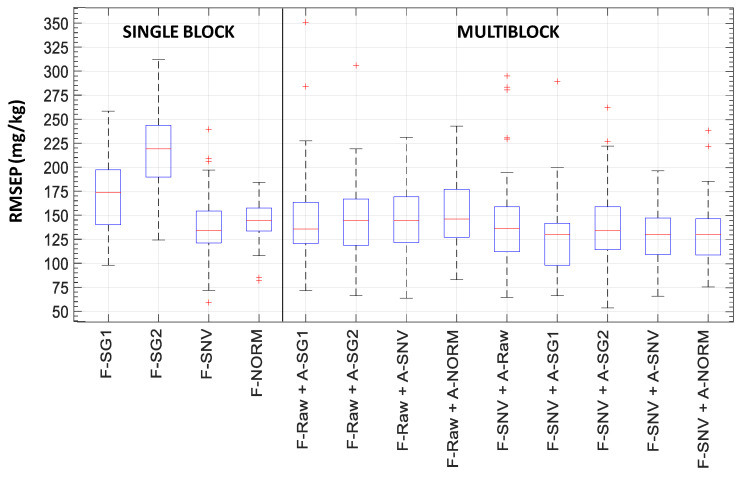
RMSEP boxplots of 50 iterations of the double cross-validation for models including pre-treated data. Bottom and top edges of the blue box are the 25th and 75th percentiles, respectively; the central mark is the median; whiskers extend to the most extreme data points not considered outliers; the ‘+’ symbol plots outliers.

**Figure 6:**
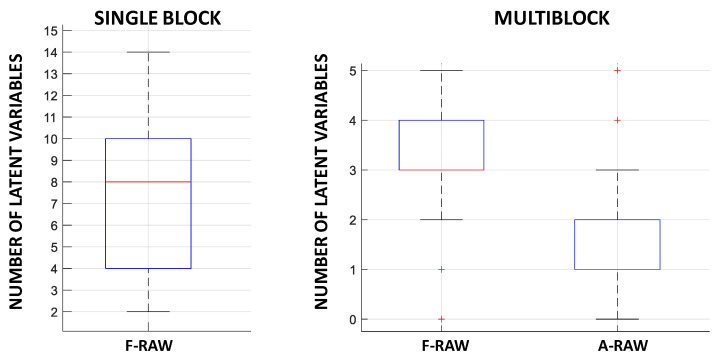
LV boxplot raw data of 50 iterations of the double cross-validation: on the left for PLSR model with fluorescence raw data; on the right for multiblock SO-PLS model with fluorescence and absorbance raw data. Bottom and top edges of the blue box are the 25th and 75th percentiles, respectively; the central mark is the median; whiskers extend to the most extreme data points not considered outliers; the ‘+’ symbol plots outliers.

Firstly, a PLSR model with double cross-validation was computed on the single block of fluorescence raw data in order to assess the capability of the emission fluorescence spectra to estimate total polyphenol content under the conditions dominated by the inner-filter effects of excitation and emission phenomena. Secondly, a model was computed by applying multiblock SO-PLS with double cross-validation in order to combine fluorescence and absorbance raw spectra. Then, a number of models were computed on single- and multiblock pretreated spectra, as detailed in Table 2. Table 3 summarizes the median values of RMSEP, the RPD computed as the STD of total polyphenols content divided by the RMSEP median, and the median values of the LV number of the 50 iterations in the double cross-validation procedure for each model. Figure 4 and Figure 5 show the RMSEP boxplots for each model. Figure 6 shows the LV boxplots for PLSR and SO-PLS models using raw spectral data.

The PLSR model on the single block of fluorescence raw spectra (F-RAW) achieved moderate performance in terms of RMSEP, with 177.11 mg·kg^−1^ as the median value. The complexity of the model was high as the median value of LVs was eight.

The combination of fluorescence and absorbance raw data provided better performance as the RMSEP median decreased to 134.45 mg·kg^−1^. Moreover, the complexity of the model was greatly reduced, which contributed to an increase in robustness. Three LVs was the median value for fluorescence data and only one LV was obtained for absorbance data instead of eight LVs when only raw fluorescence data were considered (Figure 6 and Table 3).

Figure 2 shows the regression coefficients for the F-RAW block corresponding to three LVs and the regression coefficients for the A-RAW block corresponding to one LV (c and d, respectively). Regarding F-RAW, three regions were relevant for regression coefficients. The first region ranged from 300 to 360 nm and was dominated by polyphenol fluorescence. Within this wavelength region, the F-RAW spectra presented high variability (Figure 2a). Those spectra whose emission was closer to the average spectrum seemed to show certain asymmetry.

Within the region between 400 and 600 nm, with positive regression coefficients, there was a maximum at 500 nm. The F-RAW spectra showed differences in the slope of the emission pattern around such wavelengths (Figure 2a, spanned). Lastly, the region from 600 to 781.9 nm included the dominant peak of pheophytins around 670 nm and the adjacent signal up to 720 nm where the characteristic shoulder appears, due to the reemission fluorescence of pheophytins.

Regarding the regression coefficients of the A-RAW block (Figure 2d), the values resembled the general pattern of the absorbance raw spectra, with all of them being positive. Thus, samples with higher pigment content presented higher values of the regression score computed from the absorbance data. Such scores were added to those obtained from the fluorescence data in the multiblock model applied to the estimation of polyphenol content. Those samples with higher pigment content would produce second-order inner-filter effects to a higher extent. Thus, the addition of a higher absorbance regression score would compensate for such a higher effect.

It was noticeable that, between 400 and 550 nm, the maximum regression coefficient appeared around 500 nm, which revealed that the absorbance of the carotenoids, i.e., mainly lutein in olive oil, greatly contributed to the model. Whereas information on pheophytin content was provided by both spectroscopic techniques, fluorescence and absorbance, the information on lutein was only provided by the absorbance spectra since no fluorescence has been reported for carotenoids.

These results confirm the convenience of the combination of fluorescence and absorbance spectroscopies for total polyphenol content estimation. Such a combination could provide a correction of the inner-filter effects found in the fluorescence spectra since absorbance data are related to actual pigments content involved in those inner-filter effects.

Preprocessing methods were applied to both fluorescence and absorbance spectra since they may improve the predictive ability of the linear regression models. The differentiation methods are aimed at correcting the peak overlap and additive effect, which causes a constant or linear baseline drift [16]. The Savitsky and Golay algorithm combines derivation after smoothing. The first derivative (SG1) and second derivative (SG2) were applied. Normalization is aimed at correcting for multiplicative interference, mainly due to scattering [16]. SNV and normalization by root mean square (NORM) were applied.

For a single fluorescence block, the Savitsky and Golay algorithm reduced the complexity of the model although the RMSEP median remained similar for F-SG1 and even increased for F-SG2. Such a result highlighted the fact that the fluorescence spectra showed slight deviation in the baseline (Figure 2a). On the other hand, fluorescence spectra normalized by SNV (F-SNV) provided the best model performance with the lowest RMSEP median, 134.59 mg·kg^−1^, and the lowest complexity, three LVs (see Table 3 and Figure 5). Therefore, a multiplicative effect seemed to disturb the relationship between the raw spectra and the polyphenol content.

Multiblock models with pretreated fluorescence and absorbance spectra systematically provided better performance than a single F-RAW block in terms of both lower RMSEP and lower model complexity, i.e., lower number of latent variables (Table 3 and Figure 5). The F-RAW block was combined with the different pretreated absorbance blocks. None of these combinations improved the results obtained with raw data (F-RAW + A-RAW in Figure 4 and Figure 6).

**Figure 7:**
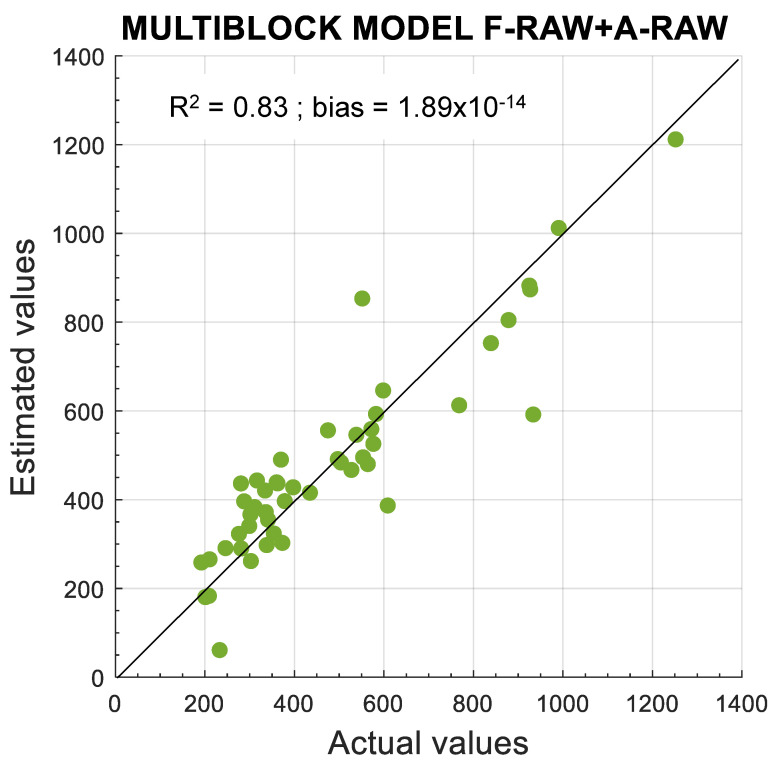
Scatter plot of actual total polyphenol content and estimated content for multiblock model using three LVs for fluorescence raw data and one LV for absorbance raw data.

In view of the good results achieved with single-block F-SNV, it was also combined with the different pretreated absorbance blocks. As shown in Table 3, the results are comparable to raw data (F-RAW + A-RAW) with a slight decrease in the RMSEP median, i.e., around 130 mg·kg^−1^, along with three LVs for F-SNV and one LV for absorbance pre-treated data. Although SNV reduces multiplicative effects and differences in the global intensities of the signals, SNV assumes that multiplicative effects are uniform over the whole spectral range, which is not always the case; thus, artefacts could be introduced by this transformation [16]. Therefore, the multiblock model using raw data for fluorescence and absorbance blocks could represent a rather valuable tool for the estimation of total polyphenol content. Figure 7 shows the scatter plot of actual total polyphenol content and estimated content for the F-RAW + A-RAW multiblock model, which achieved an *R*^2^ of 0.83.

In the present work, the Folin–Ciocâlteu analysis was carried out by an external reference laboratory specialized in the analysis of olive oil. A limitation of this method is that the phenolic compounds in olive oil include a wide range of different molecules, which could present different behavior concerning the reaction with the Folin–Ciocâlteu reagent. Phenomena of absorption and emission of light could also be affected by the relative composition of such molecules.

Despite the possible limitations, Reboredo-Rodríguez et al. [19] showed that the total phenolic content obtained by the Folin–Ciocâlteu analysis was statistically comparable to that obtained by acid hydrolysis–HPLC (as sum of hydroxytyrosol and tyrosol). The authors concluded that such analysis could be used to verify the compliance to the polyphenol health claim introduced by EU Reg. 432/2012.

Squeo et al. [6] approached the discrimination between olive oils with high and low total polyphenol content estimated by the Folin–Ciocâlteu method using fluorescence spectroscopy. This work recognized that different phenolic compounds present in olive oil have different fluorescence properties, such as spectral characteristics and fluorescence quantum yield. Nevertheless, the main conclusion was that it was possible to develop multivariate models for discrimination between oils with low and high TPC values and for quantification of TPC in olive oils, using the direct measurements of oil fluorescence. 

In any case, the problem of internal filters would persist in the fluorescence measurements and their corresponding models. Thus, the study of how to reduce their effects is a major goal, which is precisely what the present work is focused on.

This research can be part of a more complex solution. Further work is required considering a higher number of samples and cultivars, as well as the effect of the detailed composition of phenolic compounds on the estimation models based on spectroscopy.

## 4. Conclusions

The fluorescence emission spectra of olive oils acquired with excitation at 270 nm were influenced by the presence of compounds that reabsorb the light emitted by the polyphenols. In view of the wavelengths of the emission signals appearing in those spectra, compounds such as pheophytins, oxidation products, and tocopherols would absorb that light, which is known as secondary inner-filter effects. Analogously, other absorbing compounds that do not show fluorescence properties could influence the spectral pattern as well, such as luteins.

The registered characteristic emission signal of polyphenols centered at around 320 nm would be affected by the actual polyphenol content in the first instance, as well as by the actual content of pheophytins and luteins. The limited performance of the PLSR model using a single block of fluorescence spectral data (medians of 177.11 mg·kg^−1^ RMSEP and eight LVs for 50 iterations in double cross-validation) revealed that there was a deviation in the relationship between that signal and the actual polyphenol content. Moreover, those emission peaks arising from other compounds were not useful enough in the estimation model.

The combination of fluorescence emission spectra and absorbance spectra in multiblock SO-PLS models noticeably improved the estimation performance according to the decrease in RMSEP median (from 177.11 to 130.15 mg·kg^−1^) and the complexity of the model, with a lower number of LVs (from eight to four LVs). The absorbance spectra provided information on compounds responsible for inner-filter effects, both fluorophores such as pheophytins and non-fluorophores such as luteins. Hence, the absorbance spectra allowed modeling and compensating for such effects on the fluorescence data.

Combinations of pretreated fluorescence spectra by SNV normalization with different pretreated absorbance data in multiblock models achieved the best performance. Nevertheless, the simplicity and the similar results provided by the combination of fluorescence raw data and absorbance raw data in the multiblock model (medians of 134.45 mg·kg^−1^ RMSEP, three LVs for fluorescence, and one LV for absorbance) revealed their use as an easy and fast promising spectroscopic approach to the estimation of total polyphenol content.

The effect of the detailed composition of the phenolic compounds on the fluorescence and absorbance spectra, as well as on the derived estimation models, could be a major aim in further research. 

## Figures and Tables

**Table 1:** Summary of the basic statistics of reference measurements of polyphenols and pigments for the calibration and the validation sets.

Statistics (*n* = 48)	Polyphenols (mg·kg^−1^)	Pheophytins (mg·kg^−1^)	Lutein (mg·kg^−1^)
Mean	478.90	10.22	10.84
Median	375.73	7.06	7.99
STD	244.66	8.65	10.11
Min	192.00	1.97	2.84
Max	1251.00	43.75	59.51

**Table 2:** Preprocessing methods applied to fluorescence and absorbance spectral data.

Code	Preprocessing	Ref
RAW	None	[16]
SG1	First derivative by Savitsky and Golay algorithm, width 21 wavelengths, polynomial degree 3, order 1	
SG2	Second derivative by Savitsky and Golay algorithm, width 21 wavelengths, polynomial degree 3, order 2	
SNV	Standard normal variate	
NORM	Normalization by root mean square	

**Table 3:** Summary of performances of the PLSR single-block and SO-PLS multiblock models for the estimation of total polyphenol content in mg·kg^−1^ using different pretreated spectral data.

**Raw Data**				
PLSR single block	RMSEP median (mg·kg^−1^)	RPD	LV median fluorescence	
F-RAW	177.11	1.4	8	
SO-PLS multiblock	RMSEP median (mg·kg^−1^)	RPD	LV median fluorescence	LV median absorbance
F-RAW + A-RAW	134.45		3	1
**Pre-treated data**				
PLSR single block	RMSEP median (mg·kg^−1^)	RPD	LV median fluorescence	
F-SG1	174.22	1.4	3.5	
F-SG2	219.67	1.1	3	
F-SNV	134.59	1.8	3.5	
F-NORM	145.02	1.7	8	
SO-PLS multiblock	RMSEP median (mg·kg^−1^)	RPD	LV median fluorescence	LV median absorbance
F-RAW + A-SG1	136.29	1.8	3.5	2
F-RAW + A-SG2	145.12	1.7	3	2
F-RAW + A-SNV	145.23	1.7	3	1
F-RAW + A-NORM	146.6	1.7	3	2
F-SNV + A-RAW	136.87	1.8	3	1
F-SNV + A-SG1	130.15	1.9	3	1
F-SNV + A-SG2	134.4	1.8	3	1
F-SNV + A-SNV	130.31	1.9	3	1
F-SNV + A-NORM	130.37	1.9	3	1
